# Supplementary figures and images for: Effect of postmastectomy radiotherapy on T1-2N1M0 triple-negative breast cancer
Source: PLoS One. 2022 Jun 24;17(6):e0270528. doi: 10.1371/journal.pone.0270528 (PMC9231765; doi:10.1371/journal.pone.0270528)

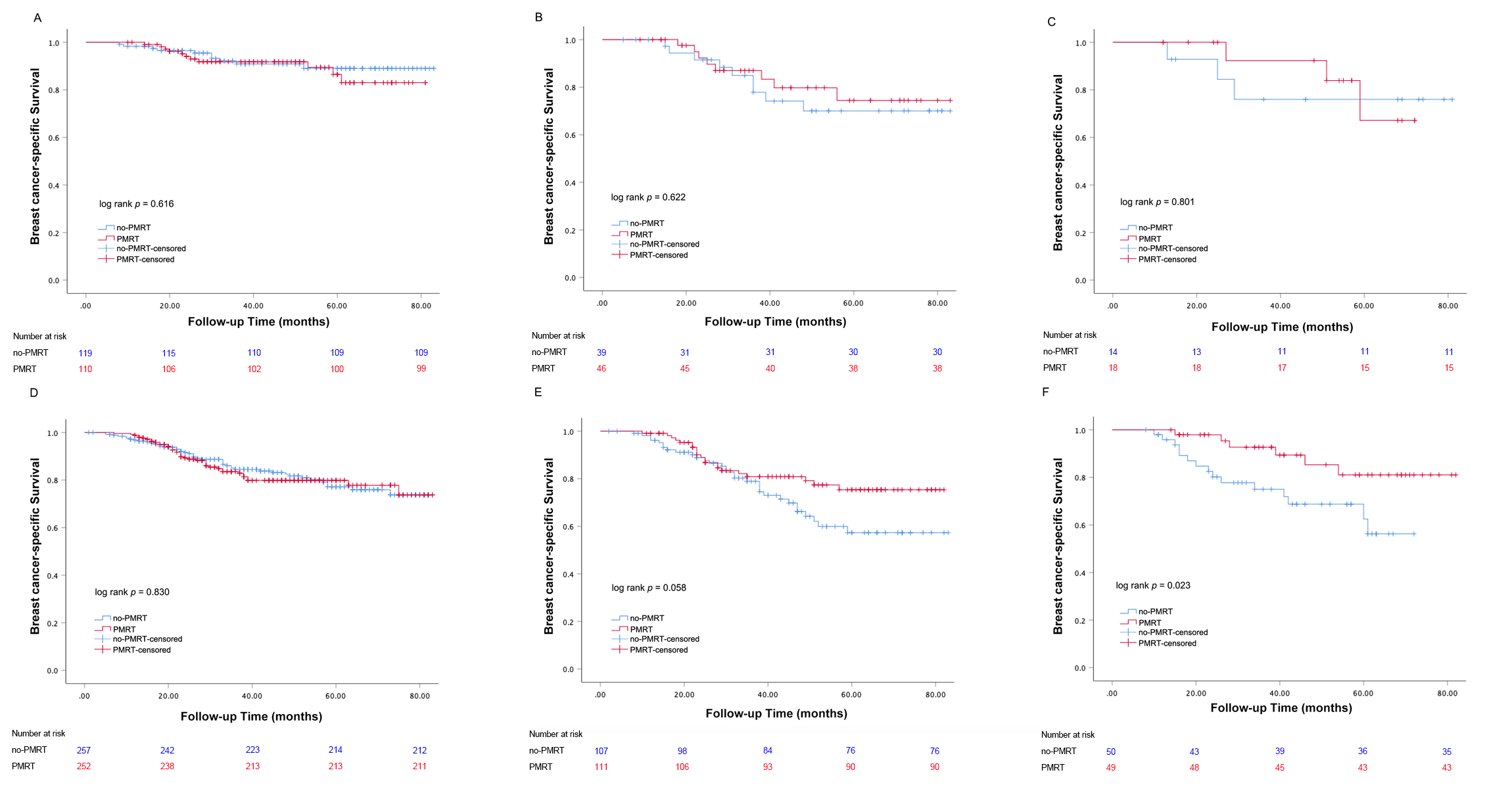

Supplement: S1 Fig — Kaplan-Meier curves of BCSS for TNBC patients with and without PMRT:(A) patients with T1 tumor and one positive LN; (B) patients with T1 tumor and two positive LNs; (C) patients with T1 tumor and three positive LNs; (D) patients with T2 tumor and one positive LN; (E) patients with T2 tumor and two positive LNs; (F) patients with T2 tumor and three positive LNs. (TIF) [file pone.0270528.s001.tif]
